# Contemporary insights into the epidemiology, impact and treatment of secondary tricuspid regurgitation across the heart failure spectrum

**DOI:** 10.1002/ejhf.2858

**Published:** 2023-05-01

**Authors:** Gregor Heitzinger, Noemi Pavo, Sophia Koschatko, Charlotte Jantsch, Max‐Paul Winter, Georg Spinka, Varius Dannenberg, Stefan Kastl, Suriya Prausmüller, Henrike Arfsten, Carolina Dona, Christian Nitsche, Kseniya Halavina, Matthias Koschutnik, Katharina Mascherbauer, Cornelia Gabler, Guido Strunk, Christian Hengstenberg, Martin Hülsmann, Philipp E. Bartko, Georg Goliasch

**Affiliations:** ^1^ Department of Internal Medicine II Medical University of Vienna Vienna Austria; ^2^ IT Systems and Communications Medical University of Vienna Vienna Austria; ^3^ Complexity Research Vienna Austria; ^4^ Department of Internal Medicine University of Szeged Szeged Hungary

**Keywords:** Secondary tricuspid regurgitation, Heart failure, HFrEF, HFpEF, HFmrEF, Transcatheter tricuspid valve intervention

## Abstract

**Aim:**

Tricuspid regurgitation secondary to heart failure (HF) is common with considerable impact on survival and hospitalization rates. Currently, insights into epidemiology, impact, and treatment of secondary tricuspid regurgitation (sTR) across the entire HF spectrum are lacking, yet are necessary for healthcare decision‐making.

**Methods and results:**

This population‐based study included data from 13 469 patients with HF and sTR from the Viennese community over a 10‐year period. The primary outcome was long‐term mortality. Overall, HF with preserved ejection fraction was the most frequent (57%, *n* = 7733) HF subtype and the burden of comorbidities was high. Severe sTR was present in 1514 patients (11%), most common among patients with HF with reduced ejection fraction (20%, *n* = 496). Mortality of patients with sTR was higher than expected survival of sex‐ and age‐matched community and independent of HF subtype (moderate sTR: hazard ratio [HR] 6.32, 95% confidence interval [CI] 5.88–6.80, *p* < 0.001; severe sTR: HR 9.04; 95% CI 8.27–9.87, *p* < 0.001). In comparison to HF and no/mild sTR patients, mortality increased for moderate sTR (HR 1.58, 95% CI 1.48–1.69, *p* < 0.001) and for severe sTR (HR 2.19, 95% CI 2.01–2.38, *p* < 0.001). This effect prevailed after multivariate adjustment and was similar across all HF subtypes. In subgroup analysis, severe sTR mortality risk was more pronounced in younger patients (<70 years). Moderate and severe sTR were rarely treated (3%, *n* = 147), despite availability of state‐of‐the‐art facilities and universal health care.

**Conclusion:**

Secondary tricuspid regurgitation is frequent, increasing with age and associated with excess mortality independent of HF subtype. Nevertheless, sTR is rarely treated surgically or percutaneously. With the projected increase in HF prevalence and population ageing, the data suggest a major burden for healthcare systems that needs to be adequately addressed. Low‐risk transcatheter treatment options may provide a suitable alternative.

## Graphical abstract

Epidemiology, impact, and treatment of secondary tricuspid regurgitation (sTR) across the heart failure (HF) spectrum in the community setting. More than moderate sTR is frequent in all HF subtypes and significantly impacts long‐term mortality. Overall treatment utilization is low despite low access burden healthcare. CI, confidence interval; HFmrEF, heart failure with mildly reduced ejection fraction; HFpEF, heart failure with preserved ejection fraction; HFrEF, heart failure with reduced ejection fraction; HR, hazard ratio; RCT, randomized controlled trial; TR, tricuspid regurgitation.

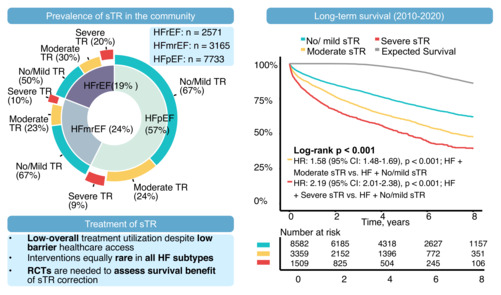

## Introduction

Tricuspid regurgitation (TR) secondary to heart failure (HF) is associated with impaired quality of life, frequent hospitalizations, and unfavourable HF trajectories.^1^, ^2^, ^3^, ^4^ Despite these adverse outcomes, secondary TR (sTR) is often perceived as an epiphenomenon exclusively reflecting the underlying ventricular disease and therefore often not mandating therapy to reduce volume overload.^5^ In addition, conservative guideline recommendations for surgical treatment reflect the associated excessive risk in these patients^6^, ^7^ due to the underlying ventricular damage, the high comorbidity burden and often systemic involvement.^8^

The true extent, impact and unmet treatment demand in sTR, however, remain unknown. Yet is crucial to assess and monitor the associated challenges for caregivers, public health authorities and service payers. Recently developed low‐risk transcatheter therapies may have the potential to address this unmet treatment demand.

In addition, there has been substantial progress in the understanding of HF and subsequently sTR. Over the past decades, definitions and diagnostic criteria have therefore significantly evolved. Recognition of subsets beyond the reduced ejection fraction range (HFrEF) as well as integration of key features beyond symptoms and ejection fraction such as natriuretic peptide activation, morphological aspects and diastolic dysfunction have reshaped and expanded these definitions to previously underrecognized or even unrecognized cohorts (i.e. those with preserved ejection fraction [HFpEF] and mildly reduced ejection fraction [HFmrEF]). Epidemiological studies of sTR have been restricted to specific subsets and therefore never examined the entire spectrum of the disease.^3^, ^9^, ^10^, ^11^ However, recent data have consistently shown that more than half of all HF patients in the general population present with HFpEF^12^, ^13^ – up to now an underrecognized part of the HF spectrum. Current knowledge on sTR epidemiology, impact and treatment standards is therefore insufficient and needs contemporary data in line with current HF definitions across the entire disease spectrum.

The aim of this study was therefore to investigate (i) demographic aspects of sTR overall and according to HF subtype, (ii) the association of sTR with mortality compared to expected survival in the age‐ and sex‐matched community, (iii) differences in subgroups, to identify patients at increased risk of mortality, and (iv) to assess treatment demand and utilization for sTR in a unique setting with a population‐wide healthcare plan and state‐of‐the‐art medical facilities.

## Methods

### Study design, clinical measures and follow‐up

In this observational study, we enrolled all individuals with HF without relevant primary valve disease in accordance with current guideline definitions from the longitudinal medical health records and echocardiography database of the Medical University of Vienna.^7^ This included all in‐ and outpatients from the Medical University of Vienna, core facilities of the Vienna healthcare alliance group with a public healthcare utility mandate for 1.5–1.9 million community residents during the study period. The definition of HF encompasses all HF subtypes. In addition to the differences between ejection fraction ranges, mandatory features for the accurate diagnosis, such as signs and symptoms, relevant structural heart disease, elevated levels of natriuretic peptides, as well as diastolic dysfunction were incorporated. *Table* *1* depicts the detailed study flow diagram as described below.

**Table 1:** Clinical, echocardiographic and laboratory parameters of patients across the heart failure spectrum according to the degree of secondary tricuspid regurgitation

	Overall (*n* = 13 469)^a^	No/mild TR (*n* = 8589)^a^	Moderate TR (*n* = 3366)^a^	Severe TR (*n* = 1514)^a^	*p*‐value*
Heart failure subtype					<0.001
HFpEF	7733 (57%)	5186 (60%)	1835 (55%)	712 (47%)	
HFmrEF	3165 (24%)	2111 (25%)	748 (22%)	306 (20%)	
HFrEF	2571 (19%)	1292 (15%)	783 (23%)	496 (33%)	
Clinical characteristics					
Male sex	8894 (66%)	6041 (70%)	2003 (60%)	850 (56%)	<0.001
Age, years	70 (61–77)	68 (59–76)	73 (66–80)	74 (66–81)	<0.001
Body mass index, kg/m^2^	27.4 (24.5–31.1)	27.8 (24.8–31.5)	26.7 (23.9–30.1)	26.3 (23.5–30.1)	<0.001
Hypertension	8224 (62%)	5342 (63%)	2081 (63%)	801 (54%)	<0.001
Hyperlipidaemia	4554 (34%)	3080 (36%)	1082 (33%)	392 (26%)	<0.001
Diabetes type II	3479 (26%)	2332 (28%)	814 (24%)	333 (22%)	<0.001
Coronary artery disease	6656 (49%)	4443 (52%)	1576 (47%)	637 (42%)	<0.001
Atrial fibrillation	4102 (31%)	2005 (24%)	1365 (41%)	732 (49%)	<0.001
Cerebral vascular disease	2413 (23%)	1494 (23%)	661 (24%)	258 (21%)	0.028
Peripheral vascular disease	3221 (25%)	2062 (25%)	808 (24%)	351 (24%)	0.7
COPD	1797 (14%)	1079 (13%)	468 (14%)	250 (17%)	<0.001
Echocardiographic characteristics					
Left ventricular end‐diastolic diameter, mm	47 (43–52)	47 (43–52)	47 (43–52)	47 (42–53)	0.5
Left ventricular end‐diastolic volume, ml	158 (124–200)	163 (129–202)	153 (121–192)	154 (114–199)	0.028
Left ventricular dysfunction					<0.001
Absent	7077 (53%)	4747 (55%)	1677 (50%)	653 (43%)	
Mild	2042 (15%)	1379 (16%)	475 (14%)	188 (12%)	
Moderate	1779 (13%)	1171 (14%)	431 (13%)	177 (12%)	
Severe	2571 (19%)	1292 (15%)	783 (23%)	496 (33%)	
Diastolic dysfunction					<0.001
Grade I	6981 (85%)	5644 (90%)	1131 (71%)	206 (50%)	
Grade II	167 (2.0%)	88 (1.4%)	61 (3.8%)	18 (4.4%)	
Grade III	1097 (13%)	506 (8.1%)	403 (25%)	188 (46%)	
Left atrial diameter, mm	58 (54–64)	57 (53–61)	61 (56–67)	65 (60–71)	<0.001
Secondary mitral regurgitation					<0.001
Mild	3863 (30%)	3271 (40%)	457 (14%)	135 (9.2%)	
Moderate	7770 (60%)	4594 (56%)	2337 (71%)	839 (57%)	
Severe	1310 (10%)	331 (4.0%)	488 (15%)	491 (34%)	
Right ventricular end‐diastolic diameter, mm	34 (30–37)	32 (30–36)	35 (31–39)	40 (35–44)	<0.001
Severe right ventricular dysfunction	2116 (16%)	592 (7.1%)	767 (23%)	757 (51%)	<0.001
Right atrial diameter, mm	57 (52–63)	55 (51–59)	60 (55–65)	66 (60–72)	<0.001
Interventricular septum, mm	13 (12–15)	14 (13–15)	13 (12–14)	13 (12–14)	<0.001
TR Vmax, m/s	3.0 (2.7–3.3)	2.8 (2.6–3.0)	3.1 (2.9–3.5)	3.4 (3.0–3.8)	<0.001
Pacemaker leads present	1342 (10%)	571 (6.6%)	459 (14%)	312 (21%)	<0.001
Pulmonary artery pressure (mmHg)	37 (30–48)	30 (30–39)	48 (41–59)	56 (46–70)	<0.001
Laboratory characteristics					
Haemoglobin, g/dl	12.8 (11.1–14.1)	13.0 (11.3–14.3)	12.4 (10.7–13.8)	12.3 (10.7–13.6)	<0.001
Red blood cell count, T/l	4.3 (3.8–4.8)	4.4 (3.9–4.8)	4.2 (3.7–4.7)	4.2 (3.7–4.7)	<0.001
Platelets, G/l	219.0 (176.0–273.0)	222.0 (179.0–276.0)	218.0 (174.0–271.0)	209.0 (165.0–261.0)	<0.001
White blood cell count, G/l	7.5 (6.0–9.4)	7.5 (6.1–9.5)	7.4 (6.0–9.2)	7.4 (5.9–9.0)	<0.001
Creatinine, mg/dl	1.05 (0.87–1.34)	1.02 (0.85–1.28)	1.08 (0.88–1.41)	1.15 (0.92–1.52)	<0.001
Blood urea nitrogen, mg/dl	18.6 (14.1–26.0)	17.6 (13.6–23.9)	19.9 (14.8–28.4)	22.5 (16.3–33.8)	<0.001
Bilirubin, mg/dl	0.6 (0.4–0.9)	0.6 (0.4–0.8)	0.7 (0.4–1.0)	0.8 (0.6–1.3)	<0.001
Albumin, g/L	38.8 (34.4–42.2)	39.3 (35.0–42.5)	38.2 (33.8–41.6)	37.7 (33.4–41.2)	<0.001
Alpha‐amylase, U/L	53 (38–74)	54 (39–74)	53 (38–74)	52 (37–74)	0.3
Cholinesterase enzyme kU/L	6.34 (4.86–7.79)	6.73 (5.28–8.13)	5.93 (4.53–7.22)	5.12 (3.94–6.46)	<0.001
Alkaline phosphatase, U/L	73 (59–95)	71 (57–90)	75 (60–98)	85 (65–116)	<0.001
Aspartate transaminase, U/L	26 (20–37)	26 (20–37)	26 (21–36)	28 (22–40)	<0.001
Alanine transaminase, U/L	24 (17–37)	25 (17–38)	23 (16–36)	23 (16–37)	<0.001
γ‐Glutamyl transferase, U/L	40.0 (23.0–81.0)	36.0 (22.0–68.0)	44.0 (24.0–90.0)	71.0 (36.0–140.0)	<0.001
Lactate dehydrogenase, U/L	213 (177–270)	206 (172–264)	220 (183–272)	234 (195–286)	<0.001
Creatine kinase, U/L	86 (53–145)	92 (55–154)	79 (49–132)	74 (47–124)	<0.001
HbA1c, %	5.9 (5.5–6.5)	5.9 (5.5–6.5)	5.8 (5.5–6.4)	6.0 (5.6–6.5)	<0.001
Total cholesterol, mg/dl	159 (129–193)	164 (133–198)	155 (126–186)	141 (114–171)	<0.001
High‐sensitivity C‐reactive protein, mg/dl	0.8 (0.2–2.6)	0.7 (0.2–2.5)	0.8 (0.3–2.8)	1.0 (0.3–2.7)	<0.001
NT‐proBNP, pg/ml	1195 (426–3404)	788 (321–2157)	2119 (812–4932)	3457 (1606–7951)	<0.001
TV treatment within the observation period					0.5
TV repair	143 (87%)	15 (88%)	46 (94%)	82 (84%)	
TV replacement	10 (6.1%)	1 (5.9%)	2 (4.1%)	7 (7.1%)	
TTVI	11 (6.7%)	1 (5.9%)	1 (2.0%)	9 (9.2%)	

COPD, chronic obstructive pulmonary disease; HbA1c, glycated haemoglobin; HFmrEF, heart failure with mildly reduced ejection fraction; HFpEF, heart failure with preserved ejection fraction; HFrEF, heart failure with reduced ejection fraction; NT‐proBNP, N‐terminal pro‐B‐type natriuretic peptide; TR, tricuspid regurgitation; TV, tricuspid valve; TTVI, transcatheter tricuspid valve intervention.

^a^ *n* (%), secondary TR severity used as denominator; median (interquartile range).

* Pearson's Chi‐squared test; Kruskal–Wallis rank sum test; Fisher's exact test.

In patients with a left ventricular ejection fraction (LVEF) >40%, at least one of the following criteria was required according to the HF guideline definitions^7^: relevant structural heart disease defined as left atrial enlargement and/or left ventricular (LV) hypertrophy, or diastolic dysfunction using recommended thresholds. Additionally, signs and symptoms of HF and an elevated N‐terminal pro‐B‐type natriuretic peptide (NT‐proBNP) >125 pg/ml were considered for the diagnosis. Patients presenting with significant primary valve disease were excluded from the study, in particular patients with evidence of (i) primary/organic tricuspid valve disease regardless of the degree of TR (myxomatous, fibroelastic deficient, or rheumatic valve disease; prolapse, flail, perforation or cleft of at least one leaflet; as well as endocarditis or congenital tricuspid valve disease and cardiovascular implantable electronic device‐related TR), (ii) any other significant primary valve disease (evidence of endocarditis/carcinoid valve disease, primary disease of the mitral valve, ≥ moderate aortic stenosis, ≥ moderate aortic regurgitation, ≥ moderate pulmonary valve stenosis or regurgitation).

Using the above outlined algorithm, 26 986 patients were excluded due to the absence of relevant structural heart disease and 4822 patients due to primary valve disease. In order to establish a reliable diagnosis of HF, the remaining patient IDs were matched with the electronic medical records to assess clinical and neurohumoral status of the patients. Subsequently, patients presenting without signs and symptoms of HF and with NT‐proBNP levels <125 pg/ml (*n* = 6843) were excluded. Furthermore, technically compromised exams with an undefinable degree of sTR or LVEF as well as focused exams without grading of sTR or LVEF were excluded (*n* = 875). The final cohort comprised 13 469 individuals and was derived from the echocardiographic database using targeted keyword searches from echocardiographic reports. In a second step, the relevant codes from the international statistical classification of diseases and related health problems were collected for the medical history and patient data were matched with the corresponding laboratory data. A custom software developed by the Medical University of Vienna was used as primary research documentation and data tool. Venous blood samples were used to analyse routine laboratory parameters according to the local laboratory's standard procedure.

All‐cause mortality was chosen as the primary endpoint and determined via retrieval query of the Austrian Death Registry. Austrian law dictates that all deaths of Austrian citizens (also in foreign countries, if reported to Austrian officials) have to be registered in the central Austrian Death Registry, allowing almost complete follow‐up of all patients. The average age‐ and sex‐matched annual mortality rates of the Austrian general population corresponding to each patient were retrieved from the Austrian life tables of 2018 provided by the Austrian Statistical Office (Statistics Austria: www.statistik.at/web_en/statistics/PeopleSociety/population). Using these mortality data, expected survival curves were generated as previously described.^14^, ^15^ The study was approved by the institutional ethics review board of the Medical University of Vienna.

### Echocardiographic examination

Comprehensive echocardiographic examinations were recorded in all patients according to the current guidelines.^6^ Commercially available equipment (Vivid E7 and E9, GE Healthcare, Chicago, IL, USA, and Acuson S2000, Siemens, Berlin, Germany) was used to perform standard transthoracic echocardiograms interpreted by board‐certified physicians. Cardiac dimensions were assessed using diameters in standard four‐ and two‐chamber views and biplane Simpson method was used to calculate LVEF. Right ventricular (RV) function was assessed quantitatively by experienced echocardiographers using multiple acoustic windows and graded with additional information from the local echo lab standard parameter (tricuspid annular plane systolic excursion, pulsed‐wave Doppler s' velocity and RV free wall strain) as mild, moderate, and severe in accordance with current recommendations.^6^, ^16^ Secondary TR was graded using multiple acoustic windows by an integrated approach. Assessment of tricuspid valve morphology, vena contracta width, hepatic flow reversal and proximal flow convergence was applied as necessary to categorize sTR into mild, moderate, moderate‐to‐severe and severe regurgitation.^2^, ^17^ Other valvular regurgitation and stenosis were graded according to societal recommendations.^6^ Pulmonary artery systolic pressures were calculated by adding the estimated central venous pressure to the peak TR systolic gradient.

### Statistical analysis

Discrete data were presented as count and percentage and compared by Fisher's exact test and Chi‐square test. Continuous data were presented as median and interquartile range (IQR) and analysed by the Kruskal–Wallis test. Cox proportional hazard regression analysis was applied to assess the impact of sTR (no/mild, moderate, severe) on survival, the results are shown as hazard ratios (HR) with 95% confidence intervals (CI). Initially, an unadjusted analysis with sTR severity as single exploratory variable was conducted. To account for potential confounding effects, adjusted multivariable analysis with a clinical confounder model (*encompassing*: age, sex, ischaemic heart disease, serum creatinine, RV end‐diastolic diameter, and RV function) and a bootstrap‐adjusted confounder model was performed.^18^ A stepwise bootstrap resampling procedure including all variables presented in *Table* *1* was used to identify best‐fitting variables for the final bootstrap model. A total of 500 repeats with a *p*‐value of 0.05 for selection were performed and variables selected in >95% of all repeats were included in the final bootstrap‐confounder model (encompassing: body mass index, diabetes, chronic obstructive pulmonary disease, peripheral vascular disease, ischaemic heart disease, LV function, blood urea nitrogen, bilirubin, albumin, and γ‐glutamyl transferase).^14^, ^19^ The proportional hazards assumption was tested and satisfied in all cases using Schoenfeld residuals. In the multivariable model, collinearity was tested using the variance inflation factor. Subgroup analysis was conducted in order to assess the impact of severe sTR on outcome in HF and across the different HF subtypes (HFpEF, HFmrEF, HFrEF). Kaplan–Meier analysis (log‐rank test) was applied to assess the time‐dependent discriminative power of sTR in HF. Two‐sided *p*‐values <0.05 were considered statistically significant. SPSS 26.0, Stata 13.0 and R‐3.6.2 were used for all statistical analyses.

## Results

### Prevalence of secondary tricuspid regurgitation in heart failure

**Figure 1:**
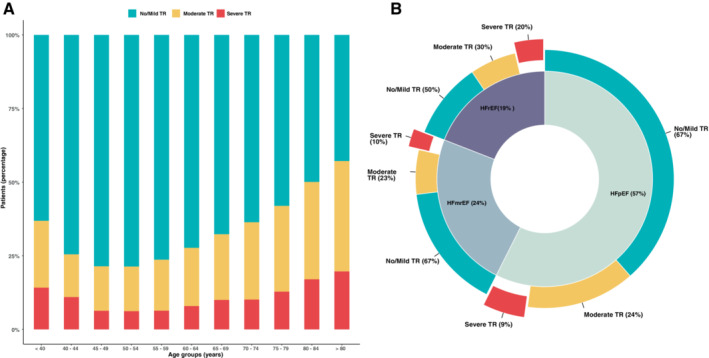
Contemporary epidemiology of secondary tricuspid regurgitation (TR): overall prevalence of no/mild, moderate and severe secondary TR stratified by age (*A*) and prevalence of no/mild, moderate, and severe secondary TR for specific heart failure subtypes (*B*). HFmrEF, heart failure with mildly reduced ejection fraction; HFpEF, heart failure with preserved ejection fraction; HFrEF, heart failure with reduced ejection fraction.

From 2010 to 2020, echocardiograms of 52 995 citizens were recorded in the longitudinal echo database. The eligibility criteria for HF according to the current guideline definitions^6^ were met by 13 469 citizens presenting without evidence of organic primary tricuspid valve disease or significant other primary valve disease. Among the total study population, sTR was absent/mild in 64% (*n* = 8589), moderate in 25% (*n* = 3366), and severe in 11% (*n* = 1514) (*Table* *1*). The majority of patients were male (66%, *n* = 8894), the median age was 70 years (IQR 61–77). Cardiovascular comorbidities were frequent: 49% (*n* = 6656) had a history of coronary artery disease, hypertension was present in 62% (*n* = 8224) and type II diabetes in 26% (*n* = 3479). Detailed baseline characteristics of the entire study population and according to TR severity grade are presented in *Table* *1*. Prevalence of more than moderate sTR also did continuously increase in patients older than 55 years (*Figure* *1A*).

With increasing sTR severity, continuously rising RV end‐diastolic diameters, RA diameters, and NT‐proBNP and creatinine levels can be observed. Severe sTR was more frequently observed in women (15%) compared to men (9.6%, *p* < 0.001). In patients with moderate or severe sTR, only 3% (*n* = 147) received treatment within the observation period, of which 2.6% (*n* = 128) underwent surgical tricuspid valve repair, 0.2% (*n* = 9) surgical tricuspid valve replacement and 0.2% (*n* = 10) transcatheter tricuspid valve interventions (TTVI). Interventions were performed equally rare in all HF subtypes (HFpEF: 1.24%, *n* = 96; HFmrEF: 1.2%, *n* = 38; HFrEF: 1.17%, *n* = 30; *p* = 0.9) (online supplementary *Figure* *S2*).

Among the 13 469 HF patients, HFpEF was present in 7733 (57%) patients, HFmrEF in 3165 (24%) and HFrEF in 2571 (19%). Severe sTR was most prevalent in HFrEF (20%, *n* = 496), followed by 10% (*n* = 306) in HFmrEF, and 9% (*n* = 712) in HFpEF (*p* < 0.001). *Figure* *1B* depicts prevalence of sTR within each HF subtype. Detailed baseline characteristics according to severity of sTR and HF type are presented in online supplementary *Tables* *S1* *–S3*.

In brief, significantly larger RV end‐diastolic diameters in HFrEF (35 mm [IQR 31–40]) and HFmrEF (34 mm [30‐37 mm]) as compared to patients with HFpEF (33 mm [IQR 30–36]; *p* < 0.001) as well as a lower prevalence of atrial fibrillation (HFrEF: 30%, HFmrEF: 29%, HFpEF 33%; *p* = 0.001) were observed. Details are presented in *Table* *1*.

### Outcome of secondary tricuspid regurgitation in heart failure

**Figure 2:**
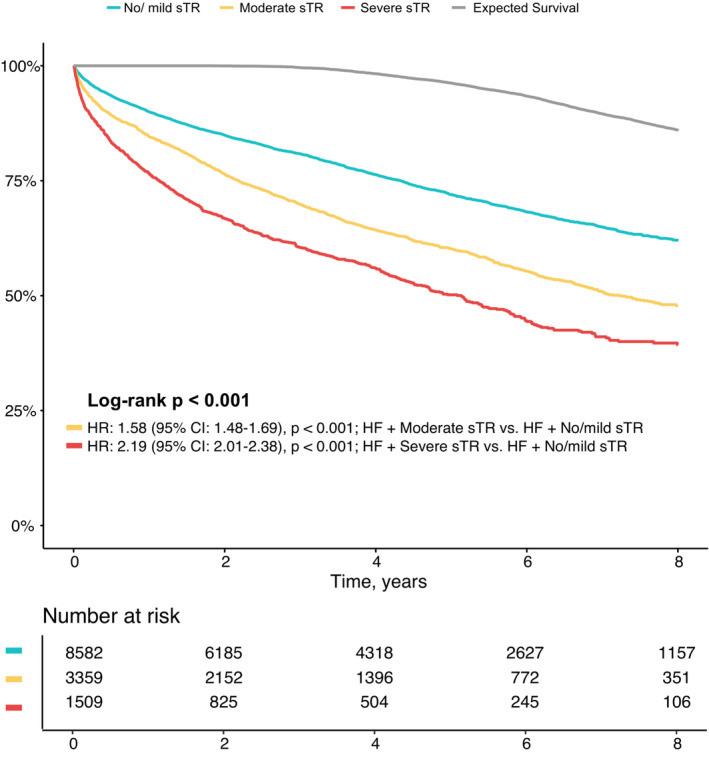
Impact of secondary tricuspid regurgitation (sTR): Kaplan–Meier survival analysis according to severity of sTR in patients with heart failure (HF). Long‐term survival analysis comparing patients with HF and no/mild, moderate, or severe sTR (log‐rank *p* < 0.001) and age‐ and sex‐matched patients for comparison (grey line). CI, confidence interval; HR, hazard ratio.

During a median follow‐up of 44 months (IQR 18–75 months), 3298 patients (27.3%) died. The proportion of observed fatal events for severe sTR at 4 years amounted to 44% and 24% for no/mild sTR, respectively, while the expected fatal event rate for age‐ and sex‐matched community was at 2%. At 8 years, severe sTR patients had an event rate of 61% (38% for no/mild sTR) as compared to expected 15% in the age‐ and sex‐matched community. Kaplan–Meier curves for each severity grade and expected survival of the age‐ and sex‐matched community are presented in *Figure* *2*. With the expected survival as reference, moderate and severe sTR in particular were associated with excess mortality (moderate: HR 6.32, 95% CI 5.88–6.80, *p* < 0.001; severe: HR 9.04, 95% CI 8.27–9.87, *p* < 0.001). When compared to patients with HF and no/mild sTR (*Table* *2*), a stepwise increase in risk dependent on sTR severity could be observed with an unadjusted HR of 1.58 (95% CI 1.48–1.69, *p* < 0.001) for moderate sTR and of 2.19 (95% CI 2.01–2.38, *p* < 0.001) for severe sTR. After adjustment for a bootstrap confounder model and a clinical confounder model, results remained similar and significant (*Table* *2*).

**Table 2:** Crude and multivariable Cox regression models assessing the impact of secondary tricuspid regurgitation on long‐term mortality

Observed sTR grade	No. of patients/events	Univariable model		Bootstrap‐adjusted confounder^a^		Clinical confounder^b^	
		Crude HR (95% CI)	*p*‐value	Adj. HR (95% CI)	*p*‐value	Adj. HR (95% CI)	*p*‐value
Total study population	13 496/4423						
No/mild sTR	8589/2369	Reference		Reference		Reference	
Moderate sTR	3366/1303	1.58 (1.48–1.69)	**<0.001**	1.35 (1.25–1.47)	**<0.001**	1.24 (1.15–1.33)	**<0.001**
Severe sTR	1514/704	2.19 (2.01–2.38)	**<0.001**	1.72 (1.56–1.90)	**<0.001**	1.52 (1.37–1.68)	**<0.001**
HFpEF	7733/2407						
No/mild sTR	5168/1453	Reference		Reference		Reference	
Moderate sTR	1835/661	1.42 (1.30–1.56)	**<0.001**	1.26 (1.13–1.40)	**<0.001**	1.18 (1.07–1.30)	**0.001**
Severe sTR	712/293	1.82 (1.60–2.06)	**<0.001**	1.51 (1.30–1.75)	**<0.001**	1.30 (1.13–1.51)	**<0.001**
HFmrEF	3242/1036						
No/mild sTR	2111/547	Reference		Reference		Reference	
Moderate sTR	748/302	1.76 (1.53–2.03)	**<0.001**	1.45 (1.23–1.72)	**<0.001**	1.28 (1.10–1.49)	**0.002**
Severe sTR	306/156	2.64 (2.21–3.16)	**<0.001**	2.22 (1.79–2.74)	**<0.001**	1.66 (1.34–2.05)	**<0.001**
HFrEF	2619/980						
No/mild sTR	1292/369	Reference		Reference		Reference	
Moderate sTR	783/340	1.75 (1.48–1.99)	**<0.001**	1.48 (1.25–1.76)	**<0.001**	1.20 (1.02–1.41)	**0.027**
Severe sTR	496/255	2.33 (1.99–2.74)	**<0.001**	1.85 (1.54–2.24)	**<0.001**	1.52 (1.25–1.84)	**<0.001**

CI, confidence interval; HFmrEF, heart failure with mildly reduced ejection fraction; HFpEF, heart failure with preserved ejection fraction; HFrEF, heart failure with reduced ejection fraction; HR, hazard ratio; sTR, secondary tricuspid regurgitation.

^a^ Adjusted for body mass index, diabetes, chronic obstructive pulmonary disease, peripheral vascular disease, ischaemic heart disease, left ventricular function, blood urea nitrogen, bilirubin, albumin, and γ‐glutamyl transferase.

^b^ Adjusted for age, sex, ischaemic heart disease, serum creatinine, right ventricular end‐diastolic diameter, and right ventricular function.

**Figure 3:**
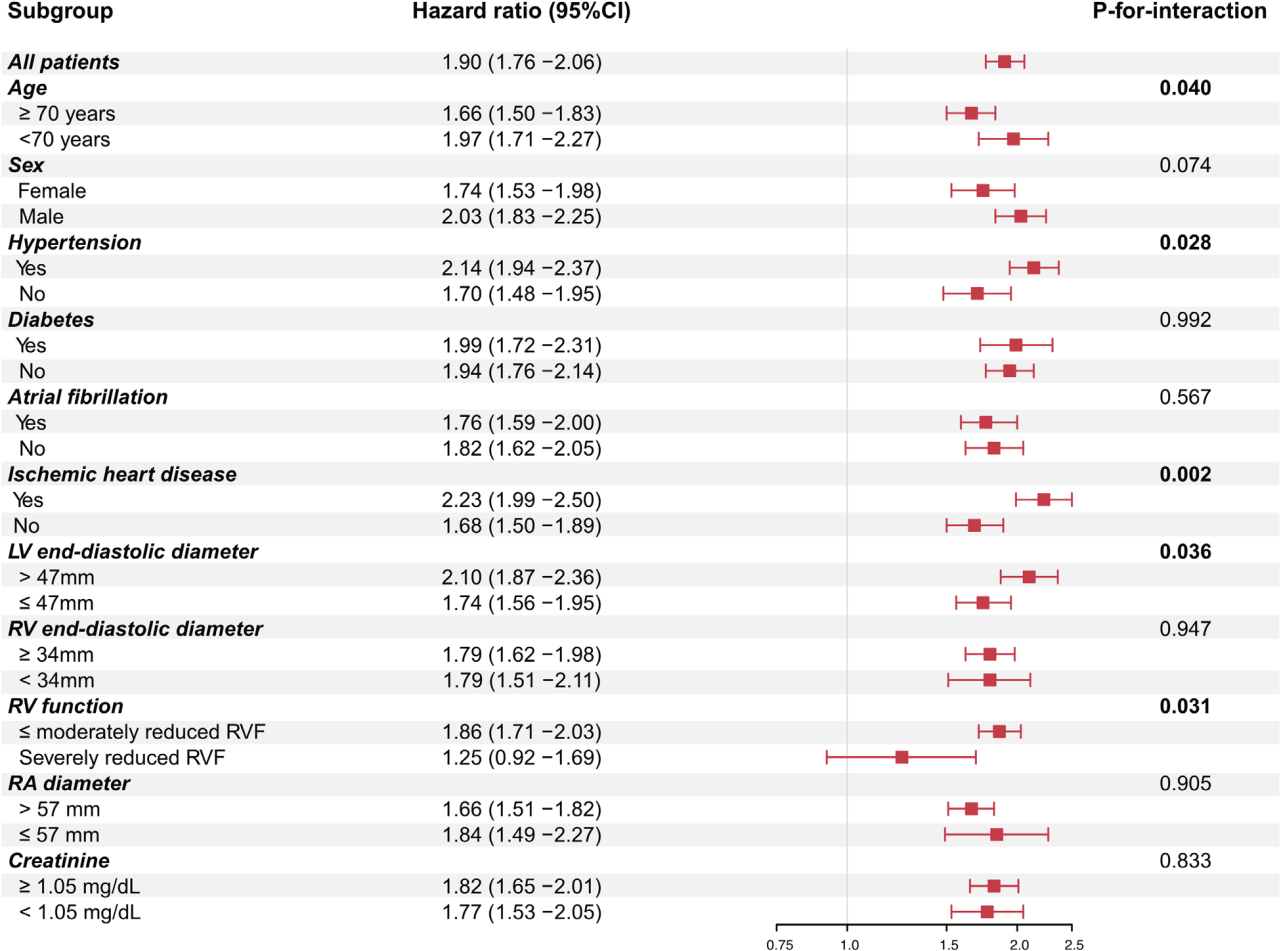
Subgroup analysis of long‐term mortality for patients with heart failure and severe secondary tricuspid regurgitation. Univariable Cox regression analyses using the median values of the total study population as cutoff points for continuous data. Severe secondary tricuspid regurgitation was a significant predictor in all subgroups, but in patients with severely reduced right ventricular function (RVF). The *p* for interaction refers to an interaction between severe secondary tricuspid regurgitation and the respective subgroup. CI, confidence interval; LV, left ventricular; RA, right atrial; RV, right ventricular.

The pronounced adverse impact of severe sTR remained in all investigated subgroups, but not for patients with severely reduced RV function (*p* = 0.155, *n* = 359) and is represented in *Figure* *3*. Significant interactions were observed between severe sTR and age, hypertension, ischaemic heart disease, LV size, and RV function. The association of severe sTR and all‐cause mortality was more pronounced in younger patients (<70 years: HR 1.97, 95% CI 1.71–2.27, *p* < 0.001; ≥70 years: HR 1.66, 95% CI 1.50–1.83, *p* < 0.001; *p* for interaction = 0.040), patients with a history of hypertension (Yes: HR 2.14, 95% CI 1.94–2.37, *p* < 0.001; No: HR 1.70, 95% CI 1.48–1.95, *p* < 0.001; *p* for interaction = 0.028), ischaemic heart disease (Yes: HR 2.23, 95% CI 1.99–2.50, *p* < 0.001; No: HR 1.68, 95% CI 1.50–1.89, *p* < 0.001; *p* for interaction = 0.002), larger LV end‐diastolic diameters (≥47 mm: HR 2.10, 95% CI 1.87–2.36, *p* < 0.001; ≤47 mm: HR 1.74, 95% CI 1.56–1.95, *p* < 0.001; *p* for interaction = 0.036) and reduced in patients with severely impaired RV function (Yes: HR 1.25, 95% CI 0.92–1.69, *p* = 0.155; No: HR 1.86, 95% CI 1.71–2.03, *p* < 0.001; *p* for interaction = 0.031).

### Outcome of secondary tricuspid regurgitation according to type of heart failure

**Figure 4:**
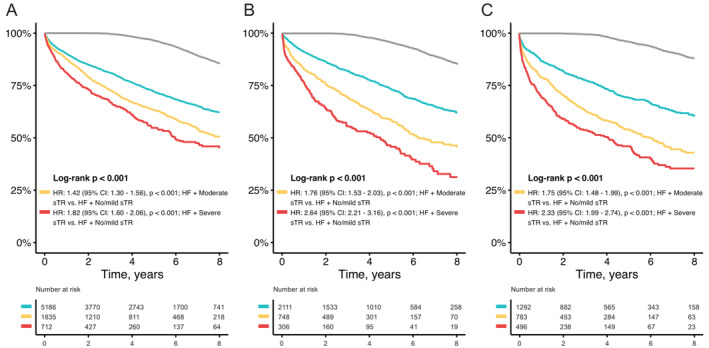
Secondary tricuspid regurgitation (sTR) across the heart failure (HF) spectrum. Long‐term survival analysis in patients with sTR (blue: no/mild sTR, yellow: moderate sTR, red: severe sTR) and age‐ and sex‐matched community for expected survival (grey line) in patients with preserved (*A*, log‐rank *p* < 0.001), mildly‐reduced (*B*, log‐rank *p* <0.001), and reduced ejection fraction (*C*, log‐rank *p* <0.001). CI, confidence interval; HR, hazard ratio.

Regardless of HF subtype, patients with no/mild sTR were already at an increased risk of mortality as compared to age‐ and sex‐matched controls (*Figure* *4*, log‐rank *p* < 0.001). Additionally, consistent with the overall analysis, the stepwise risk increase dependent on sTR severity was present across all HF subtypes (*Table* *2*) and remained after adjustment. The excessive risk of mortality in severe sTR patients was most pronounced with HFmrEF (HR 2.64, 95% CI 2.21–3.16, *p* < 0.001) followed by HFrEF (HR 2.33, 95% CI 1.99–2.74, *p* < 0.001) and HFpEF (HR 1.82, 95% CI 1.60–2.06, *p* < 0.001). Detailed results of the subgroup analysis investigating the effect of severe sTR on all‐cause mortality in HFpEF, HFmrEF and HFrEF are displayed in online supplementary *Figure* *S3–S5*. Briefly, the detrimental effect of severe sTR in patients with HFpEF, HFmrEF and HFrEF was consistent throughout all the examined subgroups.

## Discussion

The results of this study provide novel and unique insights into prevalence, outcome, and contemporary treatment of sTR across the complete spectrum of HF. For the first time these aspects are examined in a large representative population with a comprehensive HF diagnosis – consistent with current guideline criteria. These results may serve as a reference for future investigations and provide the foundation for public health monitoring, effective disease control programmes and appropriate response systems. The main findings include: (i) more than moderate sTR is highly prevalent across all HF subgroups and most common in HFrEF patients; (ii) mortality is substantial even with moderate sTR with an excess in patients with severe sTR even after adjustment for confounders; (iii) these adverse effects on survival are independently observed in all HF subtypes; (iv) despite availability of treatment options and low barrier health access, overall treatment utilization is low (Graphical Abstract).

### Epidemiological aspects to secondary tricuspid regurgitation in heart failure patients

These findings provide a unique analysis of sTR in patients with HF to further deepen the epidemiological understanding. Earlier studies^3^ focused on specific HF subtypes, thereby omitting a significant proportion of patients at high risk of mortality and limiting generalizability. In this study, severe sTR in HFpEF and HFmrEF was numerically twice as frequent as in HFrEF (*Figure* *1B*). These data indicate that the potential number of affected patients is significantly higher than previously reported and therefore needs to be accounted for by healthcare providers. Conservative estimates approximate a HF prevalence of 1–2%^20^ in the overall community. The present sample represents a considerable fraction of these 1% HF patients in the local community, in which 36% also have moderate or severe sTR, thus indicating that 2.7–5.4 million Europeans with HF may suffer from significant sTR.

Taken together with both the projected increase in an aging population and the increasing prevalence of HF,^21^ also the proportion of HF patients with concomitant sTR is likely to increase. As depicted in *Figure* *1A*, the prevalence of moderate and severe sTR continuously rises with increasing age. Previous investigations also found one third of HFrEF patients with non‐severe sTR to experience disease progression to higher severity grades.^22^ This further increases the number of patients at risk for development of more than moderate sTR that need to be accounted for. These results indicate a major burden to healthcare systems within the next decade that need to be addressed adequately by healthcare providers. Due to this cohort size and yet retained granularity, these results provide representative data that can be used for development of tailored treatment programmes and public health policy planning.

### Impact and outcome of secondary tricuspid regurgitation in heart failure

In comparison to the expected survival of an age‐ and sex‐matched standard population, patients with sTR suffer from excess mortality (*Figure* *2*). This adverse effect is already present for mild sTR (38% event rate at 8 years) indicating the HF background risk. In severe sTR, a substantially increased risk of mortality with an event rate of 61% at 8 years can be observed. Other TR and HF cohorts that have previously been reported on,^11^ show similar adverse effects of TR. These present with overall worse survival due to a higher burden of comorbidities, possibly reflecting intercontinental differences in HF patients. These detrimental effects of sTR on survival have already been described for HFrEF patients,^3^ the impact of sTR in the remaining HF subtypes has not been investigated. These results demonstrate that the unfavourable effects of sTR prevail in both HFpEF and HFmrEF. The stepwise risk increase with worsening severity was present in all HF subtypes (*Table* *2* *)*. Even after adjustment for bootstrap resampling selected and clinical confounders, the presence of moderate or severe sTR remained associated with increased mortality. Moreover, similar results were found across the entire HF spectrum, only further highlighting the detrimental effects of sTR independent of HF subtype. Additional subgroup analysis (*Figure* *3*) revealed that severe sTR also is a significant predictor of mortality in all examined subgroups, but patients with severely reduced RV function. It is likely that severe sTR contributed significantly to progression of RV disease. These results suggest a RV end‐stage phenotype where the window of opportunity for interventions has closed, and interventions are presumably futile. Therefore, timing of intervention should be carefully considered in these patients. Interestingly, this effect was independent of LV function, as severe sTR was not a significant predictor of mortality in any HF subtype for patients with severely reduced RV function (online supplementary *Figure* *S3–S5*). Additionally, the phenotype of sTR can affect survival,^23^, ^24^, ^25^ but in‐depth analysis according to HF subgroups is currently lacking.

### Current treatment standards of secondary tricuspid regurgitation

Due to a lack of data and dedicated randomized controlled data, current guidelines for the treatment of sTR remain vague and the class of recommendations are comparably low, especially in the absence of other primary valve disease requiring surgery. Addition of tricuspid valve surgery is indicated if the patient is undergoing left‐sided cardiac surgery and has severe sTR or moderate sTR and signs of RV remodelling. In patients with depressed LV function or pulmonary disease, recommendations are even more conservative. Frequently, patients with HF and sTR also suffer from high comorbidity burden and are older, both substantially increasing the surgical risk. Although in recent years several advances in the field of TTVI have been made,^26^, ^27^, ^28^, ^29^, ^30^ widespread societal recommendation is currently lacking. A recent paper investigating surgical treatment of TR in the nationwide French database, found tricuspid valve surgery to be performed infrequently and mostly with concomitant left‐sided surgery. Additionally, patients were often referred late, when RV disease has progressed, and other organ involvement has occurred. Mortality and rate of readmissions were substantial in mid‐term outcomes and related to the severity of initial presentation.^31^ The present data confirm that sTR is treated rarely. Despite availability of state‐of‐the art surgical options, adaption of transcatheter treatment techniques and a universal healthcare plan, overall treatment utilization for patients with sTR and HF remained low. This severe undertreatment is in part due to a common notion that describes TR as a ‘benign bystander disease’. On the contrary, these results provide evidence that sTR is far from benign, as already mild sTR confers a significantly increased risk of mortality. The current perception of sTR is faulted and underestimated as a separate disease, that increases mortality. Thus, it may be prudent to refer patients earlier to specialized heart valve centres and to adopt a more liberal approach regarding interventions, both surgical and transcatheter‐based.

### Implications for clinical practice

These results highlight the importance of echocardiography for diagnosis and severity assessment of sTR and HF. Echocardiography remains an efficient, cost‐effective and widespread tool to identify and monitor patients at risk. Additionally, these results indicate that early involvement of heart teams to guide the therapeutic pathway may be beneficial for improved patient management. Furthermore, randomized controlled trials are required to properly assess whether sTR correction (both surgical and transcatheter) improves patient outcomes. Recently published results of the TRILUMINATE pivotal trial show a sustained TR reduction and symptomatic benefit in patients with symptomatic severe TR that were randomized to TTVI versus guideline‐directed medical therapy alone.^30^ Survival differences between groups were not significant and the potential benefits of TTVI in symptomatic HFrEF patients remains to be investigated. Building on propensity matched data on transcatheter repair,^32^ future randomized controlled trials like the Tri.Fr study will provide further necessary insight into the conundrum of TR^33^ (NCT04646811).

### Strengths and limitations

Specific strengths of this study are the following: firstly, this database encompasses an extensive yet granular sample with individual patient data, that allows HF diagnosis and subtype ascertainment according to guideline diagnostic criteria rather than determination according to diagnostic or billing codes. Secondly, the sample size covers the entire HF spectrum and also includes large proportion of patients with no/mild or moderate sTR. Thirdly, due to almost complete follow‐up, long‐term mortality can be accurately assessed. These results provide a single‐centre experience – albeit a tertiary care centre with the largest echocardiography laboratory in Austria. However, a specific referral bias cannot be excluded, Additionally, conclusions about sTR prevalence in the general population are limited due to this study's design. Furthermore, right heart catheterization was not performed in the majority of patients as it is not standard clinical practice. Therefore, haemodynamic aspects remain to be investigated. Despite comprehensive inclusion of baseline variables, detailed information on medication was not available at study inclusion but could provide relevant insights. Patients with preserved ejection fraction, but missing NT‐proBNP values were not included. Also, structured evaluation of sTR aetiology is beyond the scope of this study and remains to be investigated. Due to the study design, advanced methods for TR severity assessment and RV function grading are unavailable yet may improve risk stratification. All‐cause mortality was chosen as an endpoint to fully reflect mortality in all subgroups. Recent insights highlight significant differences in cause of death among the HF subgroups.^21^, ^34^ Therefore, all‐cause mortality may be better suited to interpret survival than cardiovascular endpoints.

## Conclusion

To conclude, these results demonstrate that moderate and severe sTR is frequent in all HF subtypes. In addition, with increasing severity of sTR, a stepwise increase in mortality can be observed, independent of the underlying HF subtype. Already mild sTR significantly impairs survival in comparison to expected survival of age‐ and sex‐matched community. Despite high availability and low access burden healthcare plans, sTR treatment is rarely used. TTVIs emerge as viable options for patients with sTR and increased surgical risk as demand for such options is likely to increase within the near future.

### Funding

This work was supported by a grant of the Austrian Science Fund (FWF – identification number: KLI‐818B).

**Conflict of interest**: none declared.

## Supporting information


**Appendix S1.** Supporting Information.
