# Supplementary material for: Contemporary insights into the epidemiology, impact and treatment of secondary tricuspid regurgitation across the heart failure spectrum
Source: Eur J Heart Fail. 2023 May 1;25(6):857–67. doi: 10.1002/ejhf.2858 (PMC10947083; doi:10.1002/ejhf.2858)
Supplement: Supplementary file 1 — Appendix S1. Supporting Information. [file EJHF-25-857-s001.docx]

**Supplement for the manuscript “Contemporary insights into the epidemiology, impact and treatment of secondary tricuspid regurgitation across the heart failure spectrum” by Heitzinger et al.**

**Supplementary Table 1.** Clinical, echocardiographic and laboratory parameters of patients according to the degree of secondary tricuspid regurgitation in HFpEF (n= 7733).

| Characteristic | Overall, N = 7,733^1^ | No/Mild TR, N = 5,186^1^ | Moderate TR, N = 1,835^1^ | Severe TR, N = 712^1^ | p-value^2^ |
| --- | --- | --- | --- | --- | --- |
| Sex, male | 4,520 (58%) | 3,331 (64%) | 891 (49%) | 298 (42%) | <0.001 |
| Age, years | 71 (63 - 78) | 70 (61 - 76) | 74 (67 - 80) | 76 (68 - 81) | <0.001 |
| Body mass index, kg/m2 | 27.7 (24.6 - 31.4) | 28.0 (24.9 - 31.6) | 27.1 (23.9 - 30.9) | 26.5 (23.5 - 30.5) | <0.001 |
| Hypertension | 4,810 (64%) | 3,280 (65%) | 1,135 (63%) | 395 (57%) | <0.001 |
| Hyperlipidemia | 2,470 (33%) | 1,763 (35%) | 545 (30%) | 162 (23%) | <0.001 |
| Diabetes, type II | 1,889 (25%) | 1,337 (26%) | 410 (23%) | 142 (21%) | <0.001 |
| Coronary Artery Disease | 3,173 (41%) | 2,242 (43%) | 699 (38%) | 232 (33%) | <0.001 |
| Atrial fibrillation | 2,390 (33%) | 1,259 (26%) | 775 (43%) | 356 (52%) | <0.001 |
| Cerebral vascular disease | 1,359 (28%) | 906 (28%) | 346 (29%) | 107 (24%) | 0.11 |
| Peripheral vascular disease | 1,844 (25%) | 1,249 (26%) | 444 (25%) | 151 (23%) | 0.2 |
| COPD | 1,006 (14%) | 630 (13%) | 264 (15%) | 112 (17%) | 0.008 |
| Left ventricular end-diastolic diameter, mm | 45 (41 - 48) | 45 (42 - 48) | 44 (41 - 48) | 43 (39 - 47) | <0.001 |
| Left ventricular end-diastolic volume, ml | 114 (89 - 145) | 125 (92 - 155) | 110 (86 - 130) | 102 (78 - 128) | 0.011 |
| Left ventricular dysfunction | 656 (8.5%) | 439 (8.5%) | 158 (8.6%) | 59 (8.3%) | >0.9 |
| Diastolic function |  |  |  |  | <0.001 |
| Grade I | 4,587 (90%) | 3,652 (93%) | 790 (84%) | 145 (72%) |  |
| Grade II | 80 (1.6%) | 41 (1.0%) | 28 (3.0%) | 11 (5.5%) |  |
| Grade III | 410 (8.1%) | 241 (6.1%) | 124 (13%) | 45 (22%) |  |
| Left atrial diameter, mm | 58 (54 - 63) | 57 (53 - 61) | 60 (55 - 66) | 64 (59 - 71) | <0.001 |
| Secondary mitral regurgitation |  |  |  |  | <0.001 |
| Mild | 2,450 (34%) | 2,100 (44%) | 279 (16%) | 71 (11%) |  |
| Moderate | 4,428 (61%) | 2,610 (54%) | 1,354 (77%) | 464 (70%) |  |
| Severe | 329 (4.6%) | 83 (1.7%) | 118 (6.7%) | 128 (19%) |  |
| Right ventricular end-diastolic diameter, mm | 33 (30 - 36) | 32 (29 - 35) | 34 (31 - 38) | 40 (34 - 44) | <0.001 |
| Right ventricular dysfunction | 477 (6.3%) | 92 (1.8%) | 171 (9.5%) | 214 (31%) | <0.001 |
| Right atrial diameter, mm | 56 (53 - 62) | 55 (52 - 59) | 59 (55 - 65) | 66 (60 - 73) | <0.001 |
| Interventricular septum, mm | 13 (12 - 15) | 14 (13 - 15) | 13 (12 - 14) | 13 (12 - 14) | <0.001 |
| TR vmax, m/s | 2.9 (2.7 - 3.3) | 2.7 (2.6 - 3.0) | 3.1 (2.8 - 3.5) | 3.5 (3.0 - 4.0) | <0.001 |
| Pulmonary artery pressure (mmHg) | 36 (30 - 46) | 30 (30 - 39) | 48 (41 - 59) | 59 (48 - 74) | <0.001 |
| Hemoglobin, g/dl | 12.6 (10.9 - 14.0) | 12.8 (11.1 - 14.2) | 12.3 (10.7 - 13.7) | 12.0 (10.5 - 13.4) | <0.001 |
| Red blood cell count, T/l | 4.3 (3.8 - 4.7) | 4.3 (3.8 - 4.8) | 4.2 (3.7 - 4.6) | 4.2 (3.6 - 4.6) | <0.001 |
| Platelets | 221.0 (177.0 - 275.0) | 222.0 (178.0 - 278.0) | 221.0 (178.0 - 273.0) | 214.0 (169.0 - 263.2) | 0.002 |
| White blood cell count, G/l | 7.3 (5.9 - 9.1) | 7.3 (6.0 - 9.2) | 7.2 (5.8 - 8.9) | 7.2 (5.9 - 8.8) | 0.027 |
| Creatinine, mg/dl | 1.0 (0.8 - 1.3) | 1.0 (0.8 - 1.3) | 1.0 (0.8 - 1.3) | 1.1 (0.9 - 1.4) | <0.001 |
| Blood urea nitrogen, mg/dl | 17.9 (13.7 - 24.7) | 17.4 (13.4 - 23.5) | 19.0 (14.1 - 25.9) | 21.5 (15.1 - 30.9) | <0.001 |
| Bilirubin, mg/dl | 0.6 (0.4 - 0.8) | 0.6 (0.4 - 0.8) | 0.6 (0.4 - 0.9) | 0.7 (0.5 - 1.1) | <0.001 |
| Albumin, g/l | 39.2 (34.7 - 42.4) | 39.4 (35.0 - 42.5) | 38.8 (34.3 - 42.1) | 38.2 (34.0 - 41.9) | <0.001 |
| Alpha - amylase, U/l | 54 (39 - 74) | 54 (39 - 74) | 54 (39 - 74) | 53 (38 - 74) | 0.9 |
| Cholinesterase enzyme kU/l | 6.50 (5.02 - 7.90) | 6.77 (5.27 - 8.13) | 6.17 (4.79 - 7.46) | 5.53 (4.19 - 6.85) | <0.001 |
| Alcalic phosphatase, U/l | 72 (58 - 93) | 71 (57 - 90) | 74 (59 - 96) | 81 (64 - 110) | <0.001 |
| Aspartate transaminase, U/l | 25 (20 - 34) | 25 (20 - 34) | 25 (20 - 33) | 27 (21 - 36) | <0.001 |
| Alanine transaminase, U/l | 23 (16 - 34) | 23 (17 - 35) | 22 (16 - 33) | 22 (16 - 32) | <0.001 |
| Gamma-Glutamyl transferase, U/l | 36.0 (22.0 - 72.0) | 34.0 (21.0 - 65.0) | 38.0 (22.0 - 75.0) | 54.5 (32.0 - 115.0) | <0.001 |
| Lactate dehydrogenase, U/l | 207 (173 - 256) | 201 (169 - 251) | 212 (179 - 261) | 227 (192 - 273) | <0.001 |
| Creatine kinase, U/l | 82 (50 - 134) | 87 (52 - 141) | 77 (46 - 120) | 69 (42 - 118) | <0.001 |
| HbA1c, % | 5.8 (5.4 - 6.4) | 5.8 (5.4 - 6.4) | 5.8 (5.4 - 6.3) | 5.9 (5.5 - 6.4) | 0.026 |
| Total cholesterol, mg/dl | 164 (133 - 197) | 166 (135 - 201) | 161 (132 - 193) | 151 (124 - 182) | <0.001 |
| High-sensitivity C-reactive protein, mg/dl | 0.7 (0.2 - 2.2) | 0.6 (0.2 - 2.2) | 0.6 (0.2 - 2.2) | 0.8 (0.2 - 2.3) | 0.019 |
| NT-proBNP, pg/ml | 784 (334 - 2,047) | 567 (274 - 1,384) | 1,289 (545 - 2,930) | 2,180 (1,055 - 4,607) | <0.001 |
| Tricuspid Valve treatment within the observation period |  |  |  |  | 0.4 |
| TV repair | 84 (88%) | 5 (83%) | 28 (93%) | 51 (85%) |  |
| TV replacement | 7 (7.3%) | 0 (0%) | 2 (6.7%) | 5 (8.3%) |  |
| TTVI | 5 (5.2%) | 1 (17%) | 0 (0%) | 4 (6.7%) |  |
| ^1^n (%); Median (IQR) | | | | | |
| ^2^Pearson's Chi-squared test; Kruskal-Wallis rank sum test; Fisher's exact test | | | | | |

**Supplementary Table 2.** Clinical, echocardiographic and laboratory parameters of patients according to the degree of secondary tricuspid regurgitation in HFmrEF (n=3165).

| Characteristic | Overall, N = 3,165^1^ | No/Mild TR, N = 2,111^1^ | Moderate TR, N = 748^1^ | Severe TR, N = 306^1^ | p-value^2^ |
| --- | --- | --- | --- | --- | --- |
| Sex, male | 2,405 (76%) | 1,695 (80%) | 518 (69%) | 192 (63%) | <0.001 |
| Age, years | 70 (61 - 77) | 67 (58 - 75) | 74 (66 - 80) | 75 (69 - 83) | <0.001 |
| Body mass index, kg/m2 | 27.4 (24.6 - 30.7) | 28.0 (25.2 - 31.2) | 26.2 (23.9 - 29.3) | 25.8 (23.6 - 29.2) | <0.001 |
| Hypertension | 1,988 (63%) | 1,333 (63%) | 489 (65%) | 166 (54%) | 0.003 |
| Hyperlipidemia | 1,225 (39%) | 857 (41%) | 278 (37%) | 90 (29%) | <0.001 |
| Diabetes, type II | 839 (27%) | 598 (28%) | 167 (22%) | 74 (24%) | 0.004 |
| Coronary Artery Disease | 1,965 (62%) | 1,392 (66%) | 425 (57%) | 148 (48%) | <0.001 |
| Atrial fibrillation | 928 (29%) | 453 (21%) | 312 (42%) | 163 (53%) | <0.001 |
| Cerebral vascular disease | 622 (20%) | 393 (19%) | 160 (21%) | 69 (23%) | 0.11 |
| Peripheral vascular disease | 778 (25%) | 516 (24%) | 174 (23%) | 88 (29%) | 0.2 |
| COPD | 398 (13%) | 256 (12%) | 98 (13%) | 44 (14%) | 0.5 |
| Left ventricular end-diastolic diameter, mm | 49 (45 - 53) | 49 (46 - 54) | 49 (44 - 53) | 47 (43 - 51) | <0.001 |
| Left ventricular end-diastolic volume, ml | 148 (120 - 176) | 154 (128 - 185) | 138 (117 - 164) | 122 (98 - 150) | <0.001 |
| Left ventricular dysfunction |  |  |  |  | 0.5 |
| Mild | 1,386 (44%) | 940 (45%) | 317 (42%) | 129 (42%) |  |
| Moderate | 1,779 (56%) | 1,171 (55%) | 431 (58%) | 177 (58%) |  |
| Diastolic function |  |  |  |  |  |
| Grade I | 1,651 (86%) | 1,399 (92%) | 215 (64%) | 37 (56%) |  |
| Grade II | 46 (2.4%) | 27 (1.8%) | 19 (5.7%) | 0 (0%) |  |
| Grade III | 230 (12%) | 101 (6.6%) | 100 (30%) | 29 (44%) |  |
| Left atrial diameter, mm | 59 (54 - 64) | 57 (53 - 61) | 62 (57 - 67) | 66 (61 - 71) | <0.001 |
| Secondary mitral regurgitation |  |  |  |  | <0.001 |
| Mild | 622 (20%) | 554 (26%) | 54 (7.2%) | 14 (4.6%) |  |
| Moderate | 2,214 (70%) | 1,465 (69%) | 562 (75%) | 187 (61%) |  |
| Severe | 329 (10%) | 92 (4.4%) | 132 (18%) | 105 (34%) |  |
| Right ventricular end-diastolic diameter, mm | 34 (30 - 37) | 33 (30 - 36) | 35 (32 - 38) | 38 (34 - 43) | <0.001 |
| Right ventricular dysfunction | 453 (15%) | 151 (7.3%) | 155 (21%) | 147 (49%) | <0.001 |
| Right atrial diameter, mm | 56 (52 - 62) | 55 (51 - 59) | 60 (55 - 66) | 65 (61 - 72) | <0.001 |
| Interventricular septum, mm | 14 (13 - 15) | 14 (13 - 15) | 14 (13 - 15) | 13 (12 - 15) | <0.001 |
| TR vmax, m/s | 2.9 (2.7 - 3.3) | 2.8 (2.5 - 3.0) | 3.1 (2.8 - 3.5) | 3.4 (3.0 - 3.7) | <0.001 |
| Pulmonary artery pressure (mmHg) | 36 (30 - 48) | 30 (30 - 39) | 48 (41 - 57) | 56 (46 - 68) | <0.001 |
| Hemoglobin, g/dl | 12.9 (11.1 - 14.2) | 13.3 (11.5 - 14.5) | 12.0 (10.4 - 13.5) | 11.8 (10.4 - 13.4) | <0.001 |
| Red blood cell count, T/l | 4.3 (3.8 - 4.8) | 4.4 (3.9 - 4.8) | 4.1 (3.6 - 4.6) | 4.1 (3.6 - 4.5) | <0.001 |
| Platelets | 218.0 (175.0 - 271.0) | 220.0 (179.0 - 273.0) | 217.0 (170.0 - 273.0) | 203.0 (164.0 - 254.0) | 0.002 |
| White blood cell count, G/l | 7.7 (6.2 - 9.7) | 7.7 (6.2 - 9.8) | 7.6 (6.2 - 9.6) | 7.3 (6.0 - 9.1) | 0.023 |
| Creatinine, mg/dl | 1.1 (0.9 - 1.3) | 1.0 (0.9 - 1.3) | 1.1 (0.9 - 1.5) | 1.2 (0.9 - 1.5) | <0.001 |
| Blood urea nitrogen, mg/dl | 18.5 (14.1 - 25.7) | 17.6 (13.6 - 23.8) | 20.0 (15.0 - 29.8) | 21.9 (16.4 - 33.8) | <0.001 |
| Bilirubin, mg/dl | 0.6 (0.4 - 0.9) | 0.6 (0.4 - 0.9) | 0.7 (0.5 - 1.0) | 0.8 (0.5 - 1.2) | <0.001 |
| Albumin, g/l | 38.6 (34.4 - 41.9) | 39.2 (35.2 - 42.4) | 37.4 (33.0 - 40.8) | 38.0 (33.1 - 40.8) | <0.001 |
| Alpha - amylase, U/l | 54 (38 - 74) | 54 (38 - 73) | 53 (38 - 74) | 54 (38 - 77) | >0.9 |
| Cholinesterase enzyme kU/l | 6.37 (4.97 - 7.78) | 6.78 (5.44 - 8.18) | 5.76 (4.46 - 6.97) | 4.92 (3.95 - 6.17) | <0.001 |
| Alcalic phosphatase, U/l | 71 (58 - 92) | 69 (57 - 88) | 74 (59 - 96) | 86 (64 - 122) | <0.001 |
| Aspartate transaminase, U/l | 28 (21 - 42) | 28 (21 - 42) | 28 (21 - 40) | 28 (22 - 42) | 0.6 |
| Alanine transaminase, U/l | 26 (18 - 40) | 27 (18 - 41) | 25 (17 - 38) | 22 (16 - 40) | 0.004 |
| Gamma-Glutamyl transferase, U/l | 41.0 (24.0 - 76.0) | 36.0 (23.0 - 66.0) | 48.0 (26.0 - 88.0) | 67.0 (32.8 - 149.5) | <0.001 |
| Lactate dehydrogenase, U/l | 220 (180 - 296) | 213 (175 - 294) | 234 (190 - 298) | 237 (195 - 299) | <0.001 |
| Creatine kinase, U/l | 99 (59 - 175) | 107 (62 - 187) | 86 (54 - 161) | 78 (52 - 132) | <0.001 |
| HbA1c, % | 5.9 (5.5 - 6.5) | 5.9 (5.5 - 6.5) | 5.8 (5.4 - 6.5) | 5.8 (5.6 - 6.3) | 0.3 |
| Total cholesterol, mg/dl | 157 (127 - 189) | 163 (130 - 194) | 153 (126 - 181) | 137 (112 - 166) | <0.001 |
| High-sensitivity C-reactive protein, mg/dl | 0.9 (0.3 - 3.2) | 0.9 (0.3 - 3.0) | 1.0 (0.3 - 3.8) | 0.8 (0.3 - 2.9) | 0.024 |
| NT-proBNP, pg/ml | 1,689 (621 - 4,010) | 1,151 (414 - 2,961) | 3,080 (1,433 - 6,174) | 2,983 (1,686 - 7,164) | <0.001 |
| Tricuspid Valve treatment within the observation period |  |  |  |  | 0.2 |
| TV repair | 32 (84%) | 6 (100%) | 12 (100%) | 14 (70%) |  |
| TV replacement | 2 (5.3%) | 0 (0%) | 0 (0%) | 2 (10%) |  |
| TTVI | 4 (11%) | 0 (0%) | 0 (0%) | 4 (20%) |  |
| ^1^n (%); Median (IQR) | | | | | |
| ^2^Pearson's Chi-squared test; Kruskal-Wallis rank sum test; Fisher's exact test | | | | | |

**Supplementary Table 3.** Clinical, echocardiographic and laboratory parameters of patients according to the degree of secondary tricuspid regurgitation in HFrEF (n= 2571).

| Characteristic | Overall, N = 2,571^1^ | No/Mild TR, N = 1,292^1^ | Moderate TR, N = 783^1^ | Severe TR, N = 496^1^ | p-value^2^ |
| --- | --- | --- | --- | --- | --- |
| Sex, male | 1,969 (77%) | 1,015 (79%) | 594 (76%) | 360 (73%) | 0.024 |
| Age, years | 67 (57 - 75) | 64 (55 - 72) | 70 (61 - 77) | 70 (60 - 78) | <0.001 |
| Body mass index, kg/m2 | 26.7 (23.9 - 30.4) | 27.2 (24.2 - 31.2) | 26.5 (23.8 - 29.4) | 26.3 (23.6 - 29.8) | 0.001 |
| Hypertension | 1,426 (55%) | 729 (56%) | 457 (58%) | 240 (48%) | 0.001 |
| Hyperlipidemia | 859 (33%) | 460 (36%) | 259 (33%) | 140 (28%) | 0.012 |
| Diabetes, type II | 751 (29%) | 397 (31%) | 237 (30%) | 117 (24%) | 0.009 |
| Coronary Artery Disease | 1,518 (59%) | 809 (63%) | 452 (58%) | 257 (52%) | <0.001 |
| Atrial fibrillation | 784 (30%) | 293 (23%) | 278 (36%) | 213 (43%) | <0.001 |
| Cerebral vascular disease | 432 (17%) | 195 (15%) | 155 (20%) | 82 (17%) | 0.021 |
| Peripheral vascular disease | 599 (23%) | 297 (23%) | 190 (24%) | 112 (23%) | 0.7 |
| COPD | 393 (15%) | 193 (15%) | 106 (14%) | 94 (19%) | 0.028 |
| Left ventricular end-diastolic diameter, mm | 56 (50 - 62) | 57 (51 - 63) | 56 (50 - 61) | 56 (50 - 62) | 0.004 |
| Left ventricular end-diastolic volume, ml | 180 (141 - 220) | 180 (145 - 222) | 172 (140 - 220) | 180 (140 - 216) | 0.4 |
| Left ventricular dysfunction |  |  |  |  |  |
| Severe | 2,571 (100%) | 1,292 (100%) | 783 (100%) | 496 (100%) |  |
| Diastolic function |  |  |  |  | <0.001 |
| Grade I | 743 (60%) | 593 (76%) | 126 (39%) | 24 (17%) |  |
| Grade II | 41 (3.3%) | 20 (2.6%) | 14 (4.4%) | 7 (4.8%) |  |
| Grade III | 457 (37%) | 164 (21%) | 179 (56%) | 114 (79%) |  |
| Left atrial diameter, mm | 61 (55 - 67) | 57 (52 - 63) | 63 (58 - 69) | 66 (61 - 72) | <0.001 |
| Secondary mitral regurgitation |  |  |  |  | <0.001 |
| Mild | 791 (31%) | 617 (48%) | 124 (16%) | 50 (10%) |  |
| Moderate | 1,128 (44%) | 519 (40%) | 421 (54%) | 188 (38%) |  |
| Severe | 652 (25%) | 156 (12%) | 238 (30%) | 258 (52%) |  |
| Right ventricular end-diastolic diameter, mm | 35 (31 - 40) | 33 (29 - 36) | 37 (33 - 41) | 41 (37 - 45) | <0.001 |
| Right ventricular dysfunction | 1,186 (47%) | 349 (28%) | 441 (58%) | 396 (81%) | <0.001 |
| Right atrial diameter, mm | 58 (52 - 65) | 54 (49 - 60) | 60 (55 - 66) | 66 (61 - 71) | <0.001 |
| Interventricular septum, mm | 13 (11 - 14) | 13 (12 - 14) | 13 (11 - 14) | 12 (11 - 14) | <0.001 |
| TR vmax, m/s | 3.1 (2.8 - 3.5) | 2.8 (2.6 - 3.1) | 3.2 (2.9 - 3.5) | 3.3 (3.0 - 3.6) | <0.001 |
| Pulmonary artery pressure (mmHg) | 44 (30 - 54) | 30 (30 - 41) | 51 (44 - 60) | 56 (47 - 65) | <0.001 |
| Hemoglobin, g/dl | 13.1 (11.5 - 14.4) | 13.4 (11.8 - 14.6) | 12.9 (11.2 - 14.3) | 12.7 (11.3 - 14.0) | <0.001 |
| Red blood cell count, T/l | 4.4 (3.9 - 4.9) | 4.5 (4.0 - 4.9) | 4.3 (3.8 - 4.8) | 4.3 (3.9 - 4.8) | <0.001 |
| Platelets | 215.0 (175.0 - 270.0) | 222.0 (181.0 - 275.0) | 212.0 (171.0 - 265.0) | 204.0 (160.0 - 260.5) | <0.001 |
| White blood cell count, G/l | 7.8 (6.4 - 9.7) | 8.0 (6.6 - 9.9) | 7.8 (6.4 - 9.5) | 7.5 (6.1 - 9.2) | <0.001 |
| Creatinine, mg/dl | 1.1 (0.9 - 1.5) | 1.1 (0.9 - 1.4) | 1.2 (1.0 - 1.6) | 1.3 (1.0 - 1.7) | <0.001 |
| Blood urea nitrogen, mg/dl | 21.0 (15.7 - 30.8) | 18.9 (14.4 - 25.8) | 22.6 (16.7 - 32.9) | 25.8 (18.7 - 40.4) | <0.001 |
| Bilirubin, mg/dl | 0.7 (0.5 - 1.1) | 0.7 (0.5 - 0.9) | 0.8 (0.5 - 1.2) | 1.0 (0.7 - 1.6) | <0.001 |
| Albumin, g/l | 38.0 (33.8 - 41.7) | 39.1 (34.7 - 42.6) | 37.5 (33.5 - 41.1) | 36.7 (33.0 - 40.1) | <0.001 |
| Alpha - amylase, U/l | 53 (38 - 74) | 53 (39 - 74) | 53 (38 - 74) | 49 (36 - 71) | 0.084 |
| Cholinesterase enzyme kU/l | 5.73 (4.30 - 7.34) | 6.45 (4.99 - 8.08) | 5.42 (4.18 - 6.88) | 4.59 (3.55 - 5.83) | <0.001 |
| Alcalic phosphatase, U/l | 78 (61 - 102) | 72 (58 - 93) | 80 (63 - 104) | 94 (70 - 122) | <0.001 |
| Aspartate transaminase, U/l | 29 (22 - 42) | 28 (21 - 42) | 28 (21 - 41) | 31 (24 - 45) | <0.001 |
| Alanine transaminase, U/l | 28 (18 - 46) | 29 (19 - 46) | 26 (17 - 42) | 28 (18 - 50) | 0.007 |
| Gamma-Glutamyl transferase, U/l | 59.0 (31.0 - 118.0) | 44.0 (25.0 - 88.0) | 65.0 (35.0 - 124.0) | 101.0 (57.0 - 172.5) | <0.001 |
| Lactate dehydrogenase, U/l | 226 (184 - 295) | 215 (176 - 292) | 230 (189 - 290) | 244 (200 - 307) | <0.001 |
| Creatine kinase, U/l | 86 (54 - 148) | 94 (57 - 164) | 80 (50 - 139) | 81 (52 - 135) | <0.001 |
| HbA1c, % | 6.0 (5.6 - 6.8) | 6.0 (5.5 - 6.8) | 5.9 (5.6 - 6.7) | 6.1 (5.7 - 6.8) | 0.051 |
| Total cholesterol, mg/dl | 147 (119 - 180) | 158 (129 - 192) | 145 (119 - 174) | 127 (105 - 158) | <0.001 |
| High-sensitivity C-reactive protein, mg/dl | 1.0 (0.3 - 3.1) | 0.8 (0.3 - 3.1) | 1.2 (0.3 - 3.2) | 1.3 (0.5 - 3.0) | 0.001 |
| NT-proBNP, pg/ml | 3,721 (1,582 - 8,572) | 2,200 (949 - 5,146) | 4,969 (2,383 - 12,000) | 6,761 (3,842 - 14,918) | <0.001 |
| Tricuspid Valve treatment within the observation period |  |  |  |  | 0.3 |
| TV repair | 27 (90%) | 4 (80%) | 6 (86%) | 17 (94%) |  |
| TV replacement | 1 (3.3%) | 1 (20%) | 0 (0%) | 0 (0%) |  |
| TTVI | 2 (6.7%) | 0 (0%) | 1 (14%) | 1 (5.6%) |  |
| ^1^n (%); Median (IQR) | | | | | |
| ^2^Pearson's Chi-squared test; Kruskal-Wallis rank sum test; Fisher's exact test | | | | | |

**Supplementary Figure 1:** Study population flow diagram: 52995 patients were screened, 4822 were excluded due to primary valve disease, 26986 did not meet diagnostic heart failure criteria. The final study population included 13469 individual patients with diagnostic heart failure and without primary valve disease. Severe sTR was present in 1514 patients (11%)


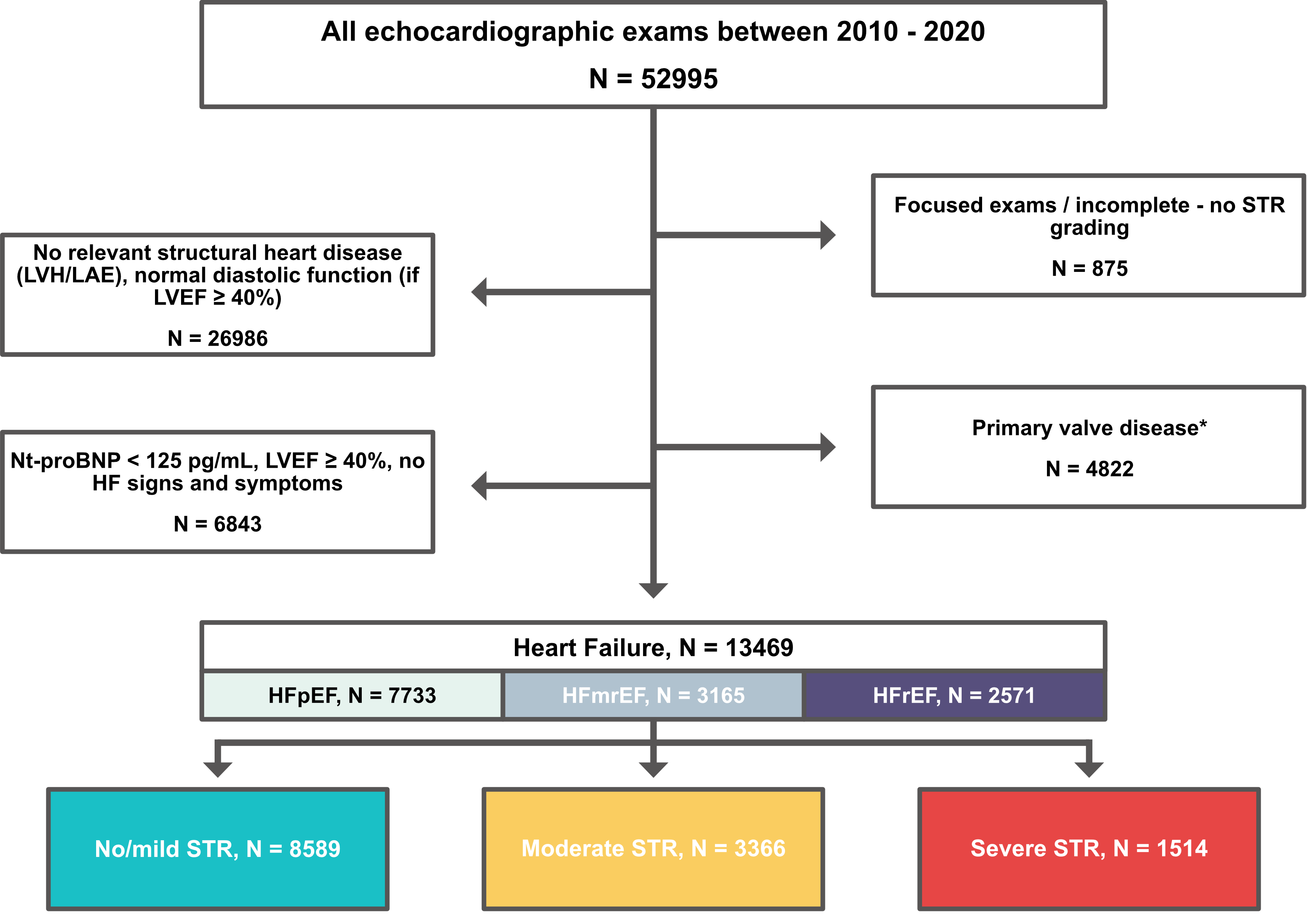


**Supplementary Figure 2:** Undertreatment of tricuspid regurgitation according to (A) sTR severity grade and (B) according to HF subgroup

**
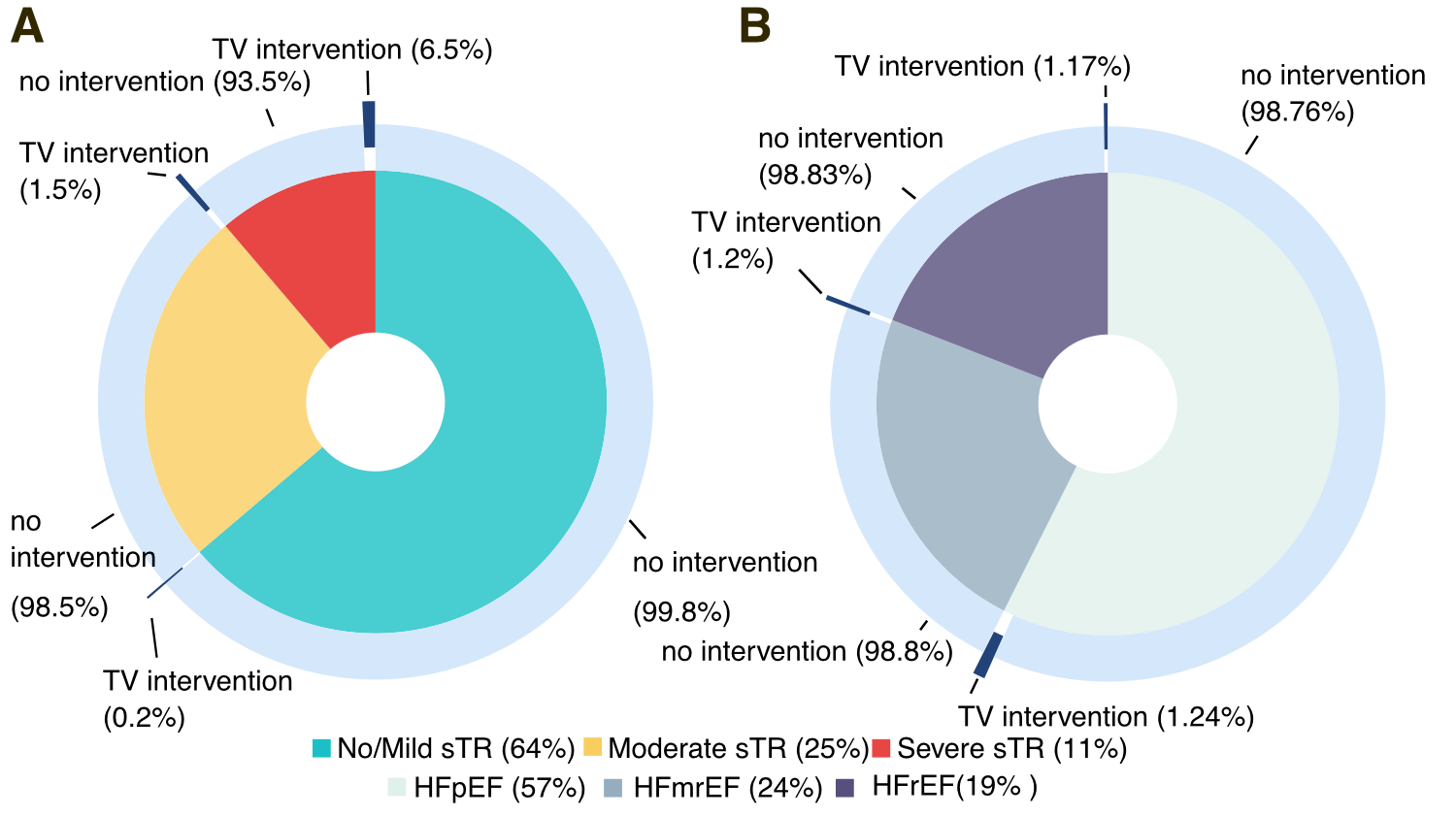
**


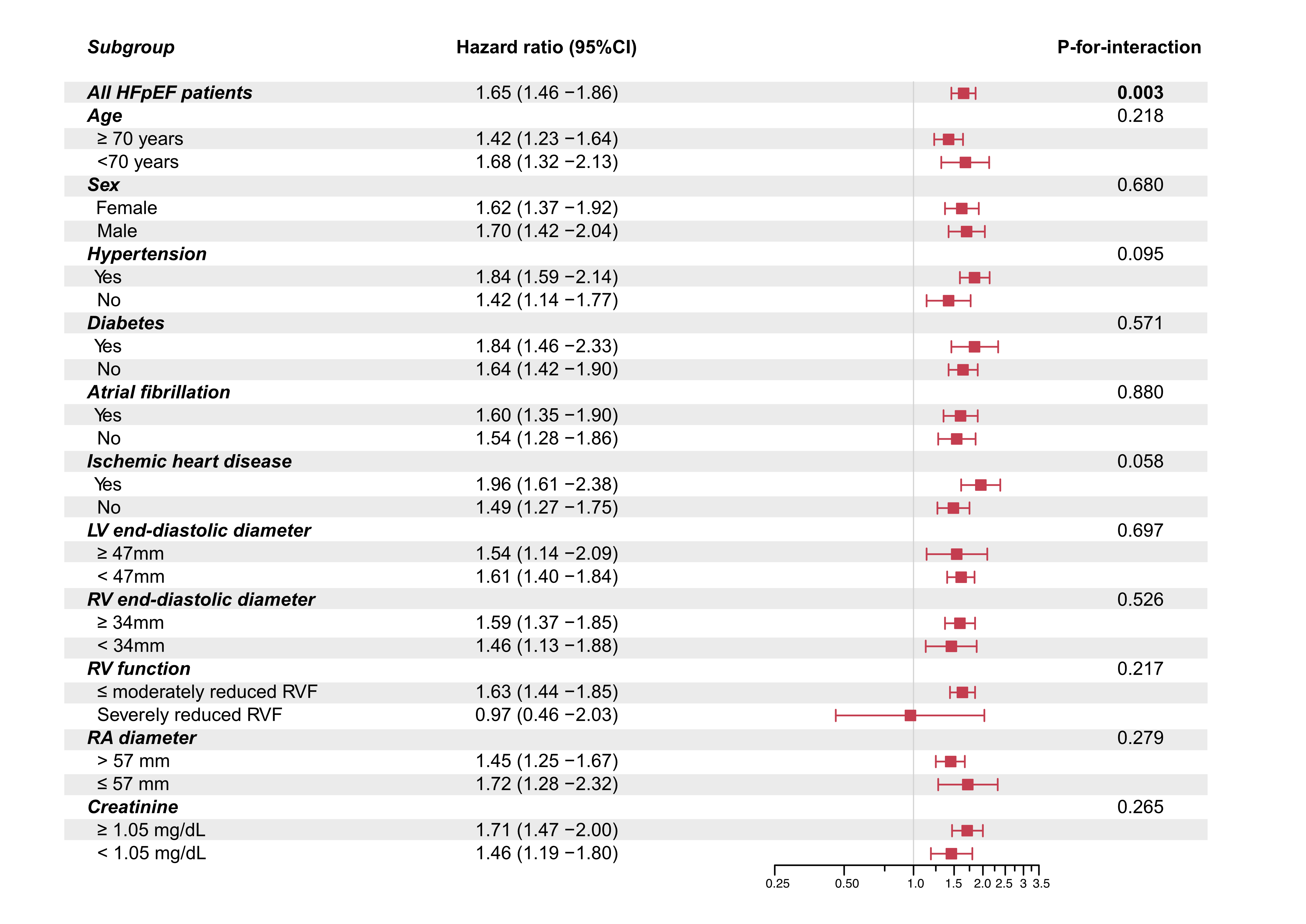
**Supplementary Figure 3:** Subgroup analysis of long-term mortality in all patients with severe sTR and HFpEF. Severe sTR was a significant predictor of mortality in all investigated subgroups, but patients with severely reduced RVF. CI=confidence interval; LA=left atrium; LV=left ventricle; RA=right atrial, RV=right ventricle; RVF=right ventricular function


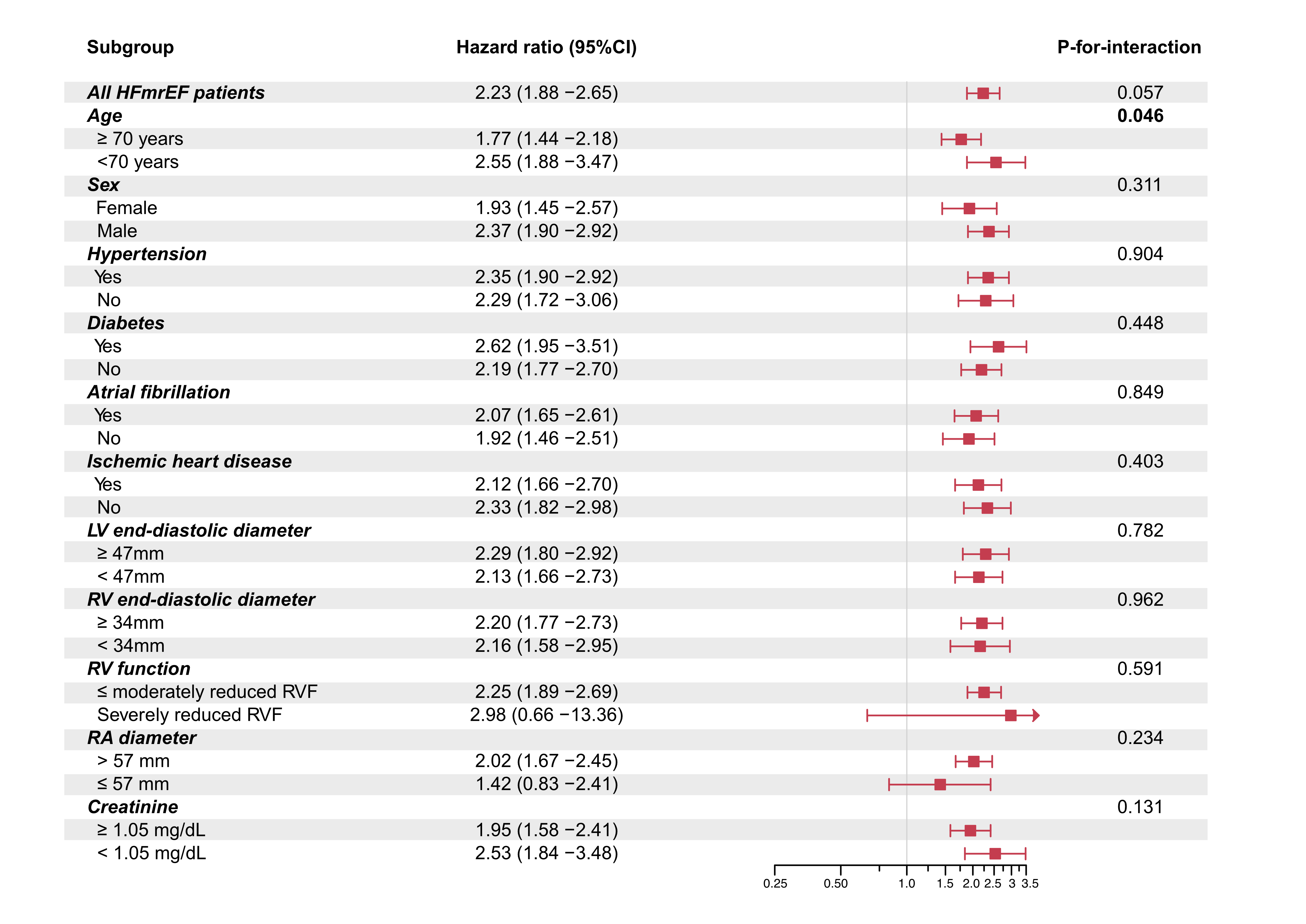
**Supplementary Figure 4:** Subgroup analysis of long-term mortality in all patients with severe sTR and HFmrEF. Severe sTR was a significant predictor of mortality in all investigated subgroups, but patients with severely reduced RVF and without severe right atrial remodeling. CI=confidence interval; LA=left atrium; LV=left ventricle; RA=right atrial, RV=right ventricle; RVF=right ventricular function

**
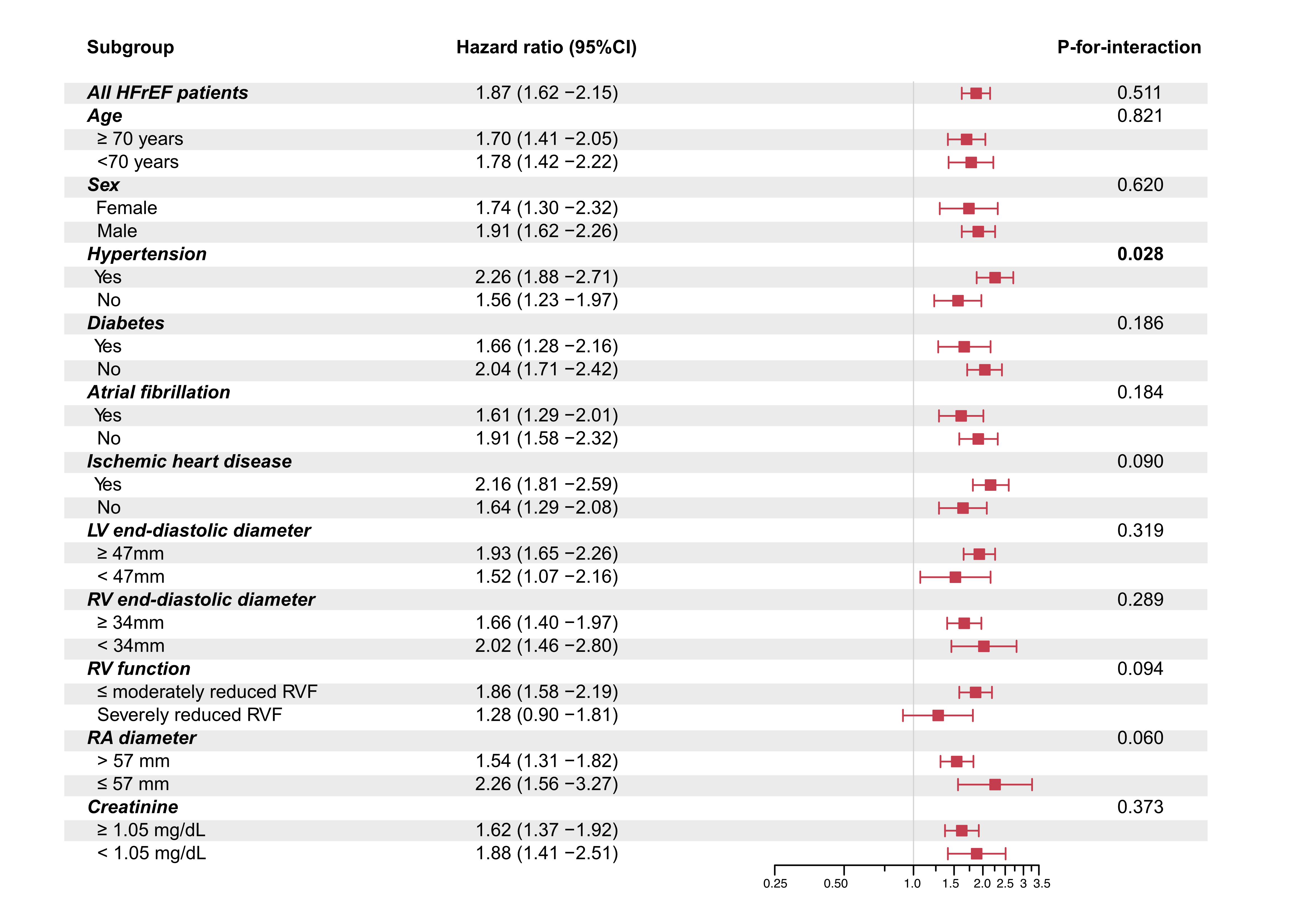
Supplementary Figure 5:** Subgroup analysis of long-term mortality in all patients with severe sTR and HFrEF. Severe sTR was a significant predictor of mortality in all investigated subgroups, but patients with severely reduced RVF. CI=confidence interval; LA=left atrium; LV=left ventricle; RA=right atrial, RV=right ventricle; RVF=right ventricular function
